# A Study Protocol for a Randomized, Double-Blind, Placebo-Controlled Clinical Study on the Effect of Qishen Yiqi Dripping Pills on Exercise Endurance and Quality of Life in Patients with Coronary Heart Disease after Percutaneous Coronary Intervention

**DOI:** 10.1155/2021/7439852

**Published:** 2021-08-24

**Authors:** Linghua Yu, Xiaoyan Lu, Xianlun Li, Hong Jiang, Ruihua Sun, Gang Chen, Cheng Xiao

**Affiliations:** ^1^China-Japan Friendship Hospital, Beijing, China; ^2^Beijing University of Traditional Chinese Medicine, Beijing, China

## Abstract

**Background:**

Percutaneous coronary intervention (PCI) is widely used in China, but it does not fundamentally improve exercise endurance or reduce mortality associated with cardiovascular disease. Standardized cardiac rehabilitation (CR) can reduce the mortality associated with coronary heart disease and reduce the need for repeated PCI procedures. Currently, research on CR after PCI is mainly based on traditional exercise prescription, while research on TCM is limited. Often, the combination of traditional Chinese medicine (TCM) and exercise rehabilitation is adopted, from which it is difficult to determine the unique advantages of TCM. Qishen Yiqi dripping pills (QSYQ) can improve myocardial energy metabolism and alleviate myocardial reperfusion injury after PCI. This paper describes the protocol for the clinical assessment of QSYQ on CR.

**Methods:**

A randomized, double-blind, placebo-controlled trial will be used to evaluate the efficacy and safety of QSYQ on improving exercise endurance and quality of life. We plan to recruit 66 patients with stable angina pectoris with Qi deficiency and blood stasis syndrome differentiation after PCI from the China-Japan Friendship Hospital. On the basis of conventional drug treatment, QSYQ or placebo will be used for 12 weeks. PeakVO_2_ will be the main efficacy evaluation index, while Seattle scale and quality of life scale will be the secondary efficacy evaluation indexes. *Discussion*. CR therapy with integrated traditional Chinese and Western medicine has been developed as a treatment modality in China and has been included in the expert consensus of TCM diagnosis and treatment. A rigorous trial design will ensure objective and scientific evaluation of the efficacy and safety of QSYQ in improving exercise endurance and quality of life in patients with PCI. *Trial Registration*. This trial is registered with Clinical trial registration in China: ChiCTR2000040838 (registration date: December 11, 2020).

## 1. Introduction

Percutaneous coronary intervention (PCI) is the most important revascularization method for patients with coronary heart disease and is widely used in clinical practice [1]. However, several issues remain, including decreased exercise endurance and decreased quality of life. Moreover, despite rigorous pharmacological interventions in patients after PCI [2], the recurrence rate of cardiovascular events is as high as 34%. Therefore, the importance of effective cardiac rehabilitation (CR) of patients after PCI cannot be ignored. Previous studies in China [3, 4] have shown that CR can effectively reduce the total mortality rate, cardiovascular mortality rate, and rehospitalization and improve the quality of life. Integrated Chinese and Western medicine treatment after PCI is a common and effective CR mode [5].

According to traditional Chinese medicine (TCM), “blood stasis syndrome” is the most common cause of coronary heart disease angina pectoris and occurs throughout the development of coronary heart disease [6]. According to the research of Professor Han's team, “Qi deficiency and blood stasis” belong to “original deficiency and standard solid” and are the core pathogenesis of reperfusion injury, myocardial hypertrophy, and chronic heart failure after interventional surgery [7]. Although interventional surgery can improve the blood supply to the heart, it does not change the “standard” and cannot improve the overall state of “blood stasis.” Additionally, from the perspective of TCM, interventional surgery belongs to mechanical and exogenous injury, which can destroy Healthy Qi and cause damage to the veins [8]. According to the theory of TCM, Qi and blood run through the pulse. If Qi is insufficient, it will affect the blood circulation and lead to blood stasis. Blood stasis will also affect the generation of Qi, leading to Qi deficiency. Qi deficiency and blood stasis interact, making the condition complex. This situation complicates the pathogenesis and increases the severity of the condition.

Qishen Yiqi dripping pills (QSYQ) were approved by the China Food and Drug Administration in 2003 for treating angina pectoris of coronary heart disease (approval number: YBZ04332003-2008Z). QSYQ is a TCM preparation developed by Tasly Pharmaceutical Group Co., Ltd., which functions to invigorate Qi, relieve pain, and promote blood circulation. The pills are composed of *Astragalus membranaceus*, *Salvia miltiorrhiza*, Panax notoginseng, and Dalbergia odorifera oil. Astragaloside IV inhibits abnormal energy metabolism pathways; Danshensu inhibits oxidative stress injury; notoginseng R1 partially improves myocardial energy metabolism and inhibits oxidative stress injury; and *Dalbergia odorifera* oil upregulates CPT1A. Multitarget comprehensive intervention in the above four components improves myocardial injury and inhibits myocardial fibrosis [7]. A large multicenter clinical study showed that Qishen Yiqi dripping pills had clinical efficacy in the secondary prevention of coronary heart disease [9]. Moreover, experimental studies [10] showed that QSYQ could effectively reduce the damage to myocardial cells caused by ischemia reperfusion and protect the myocardium. Studies have also found [11] that QSYQ can effectively improve the level of serum inflammatory factors in patients after PCI, increase left ventricular ejection fraction (LVEF) and cardiac output, reduce the volume at the end of the contraction and diastolic period, and reduce the recurrence of cardiovascular events.

At present, the combination of TCM and exercise prescription is often adopted, in which it is difficult to determine the unique advantages of TCM. However, there are few studies on TCM alone. Additionally, the evaluation of sports endurance rarely uses the gold standard, such as peak oxygen consumption (PeakVO_2_) and anaerobic metabolic threshold oxygen consumption (VO_2_AT) [12, 13]. Particularly, no clinical study of TCM compounds using the combination of disease and syndrome and randomized double-blind placebo control has yet been performed.

Therefore, we designed a randomized, double-blind, placebo-controlled clinical trial to evaluate the effects of QSYQ on exercise endurance and quality of life in patients with coronary heart disease after interventional surgery. To this end, we used the peak oxygen consumption in a cardiopulmonary exercise trial as the main efficacy evaluation.

## 2. Methods/Design

### 2.1. Objectives

The application of Qishen Yiqi dripping pills can improve the exercise endurance of patients with coronary heart disease after interventional therapy, improve cardiac function, reduce recurrence of angina pectoris, and improve the quality of life of patients.

### 2.2. Design and Settings

**Figure 1:**
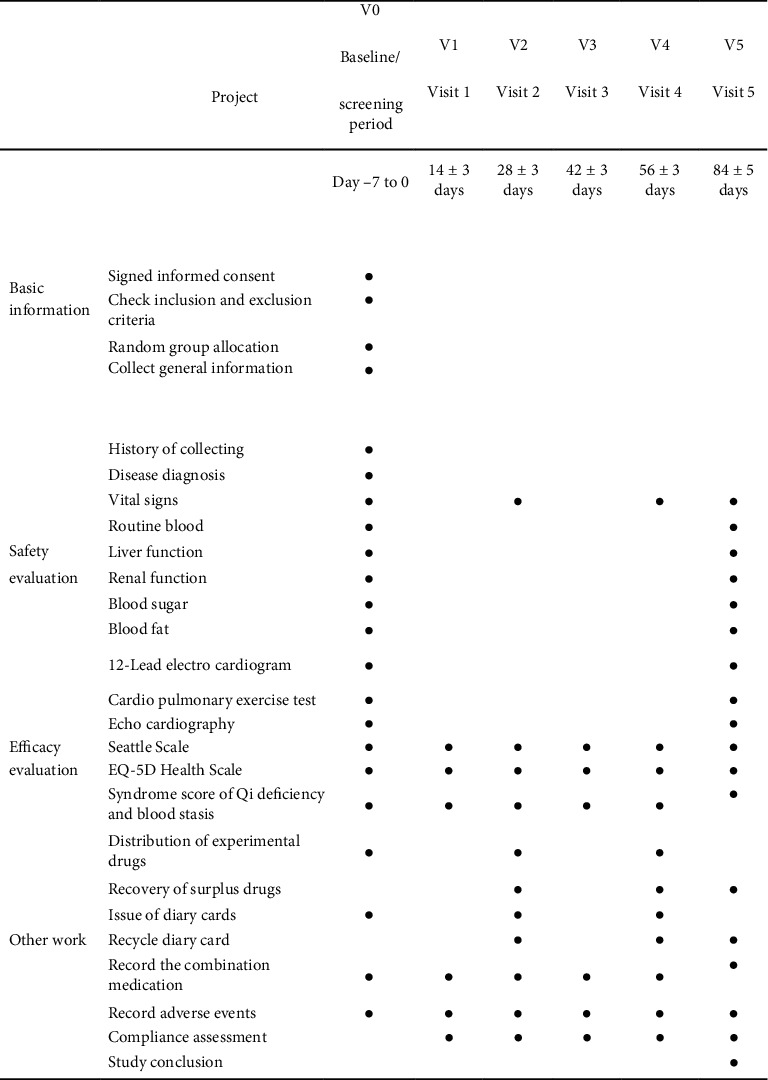
Research flowchart.

**Figure 2:**
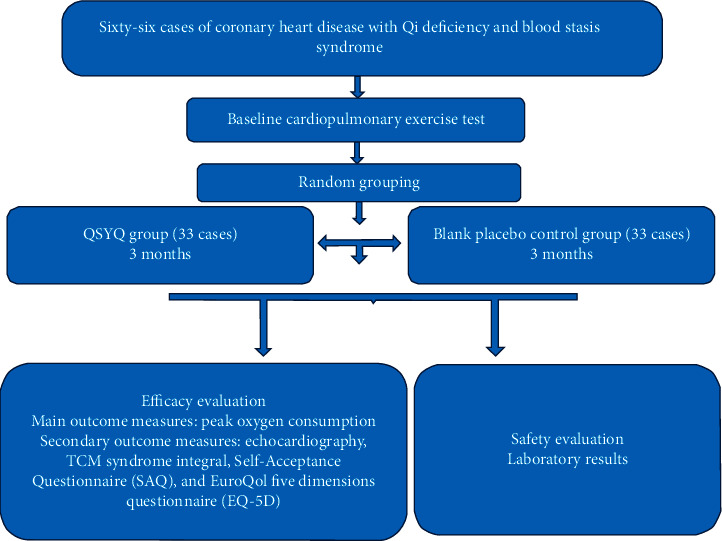
Research technology roadmap.

This trial is a randomized, double-blind, placebo-controlled clinical study, with 66 subjects planned to be enrolled. Informed consent will be signed after patients are screened by inclusion and exclusion criteria. The researchers will randomly divide the subjects into the QSYQ group and the blank placebo control group according to the time of enrollment. Before enrollment, there will be a 1-week screening period, during which other TCM drugs for CHD will be stopped. After entering the group, the patients will receive 12 weeks of medication. The flowchart of the research process is shown in Figure 1. All subjects will be followed up every 2 weeks after enrollment and will be monitored and evaluated at each follow-up. The technology roadmap is shown in Figure 2.

### 2.3. Participant Recruitment

The subjects will be recruited both on and offline. Patients who are interested in participating will decide whether to participate after being informed of the study content and purpose. The patients will be screened by the researcher according to the inclusion and exclusion criteria. Patients with “definite diagnosis of coronary heart disease,” “successful completion of interventional therapy,” and “syndrome differentiation of Qi deficiency and blood stasis after interventional therapy” will be allowed to participate.

### 2.4. Study Population

#### 2.4.1. Diagnostic Criteria

The criteria for successful intervention are as follows [14]: (1) successful angiography, in which the lumen of the coronary artery target site is significantly enlarged, with residual stenosis <50%, and achievement of TIMI-level 3 blood flow; (2) successful operation, in which the standard of successful angiography was achieved, with no major clinical complications (such as death, myocardial infarction, and emergency coronary artery bypass grafting) during hospitalization.

Diagnostic criteria of Western medicine: diagnostic criteria were devised by referring to the 2007 Guidelines for the Diagnosis and Treatment of Chronic Stable Angina Pectoris [15] and the 2016 Guidelines for the Diagnosis and Treatment of Non-ST Segment Elevation Acute Coronary Syndromes [16].

Diagnostic criteria of Qi deficiency and blood stasis syndrome: the criteria were formulated according to the Study on Syndrome Differentiation Criteria of Coronary Heart Disease after Vascular Reconstruction [17], as shown in Table 1.

#### 2.4.2. Inclusion Criteria

Inclusion criteria were as follows:

- Patients with a clear understanding of the content and purpose of the study, who will participate in the study voluntarily, and sign the informed consent
- Conformed to the diagnostic criteria of Western medicine for stable coronary heart disease
- Patients with Qi deficiency and blood stasis syndrome who meet the abovementioned TCM diagnostic criteria
- Complete the overall treatment plan after PCI
- Postcoronary heart disease intervention (1–6 months)
- Age 18–75 years
- Postcoronary heart disease intervention (1–6 months)

#### 2.4.3. Exclusion Criteria

Exclusion criteria were as follows:

- Contraindications to cardiopulmonary exercise test
- Planned coronary artery bypass grafting or heart transplant
- Stroke or transient ischemic attack occurred within 3 months before enrollment; carotid or other large vessel surgery; persistent ventricular tachycardia or ventricular fibrillation
- Ventricular arrhythmia not effectively controlled with use of antiarrhythmic drugs or implanted defibrillators
- Uncorrected primary obstructive or severe reflux valvular disease, or nondilated (restrictive) or hypertrophic cardiomyopathy
- Second- or third-degree heart block or pathological sinoatrial syndrome, without permanent pacemaker, and patients who require implantable device treatment due to heart failure
- Obstructive or bronchospasmodic lung disease (such as asthma or bronchitis) requiring oral or inhaled bronchodilators or hormone treatment
- Females who are pregnant or lactating, or who plan to become pregnant during the trial
- Cancer or other systemic disease with an expected survival of <12 months
- Use of other clinical trial drugs or have participated in medical device trials in the 30 days prior to enrollment
- Deemed unable to participate in the study after clinical evaluation

#### 2.4.4. Shedding and Selection Criteria

Shedding and Selection criteria were as follows:

- No index of peak oxygen consumption during cardiopulmonary exercise was obtained at the end of the experiment
- If a serious adverse event occurs, the researcher determines that it is necessary to withdraw the patient from the trial in advance
- Drug treatment compliance during maintenance period <80%
- The treatment process of the subjects seriously deviated from the study protocol

### 2.5. Randomization, Allocation Concealment Mechanism, and Blinding

#### 2.5.1. Random Allocation

Randomization of subjects: studies have shown that the number of diseased vessels and complicated lesions (hypertension, diabetes, and other chronic diseases) are independent risk factors affecting the recurrence of cardiovascular events after PCI and significantly influence prognosis [18]. To reduce selection bias and balance various non-study factors between groups, two factors affecting the prognosis of interventional surgery were set in this study [19–21]: (1) sex, male or female and (2) number of diseased vessels, single-, double-, or multiple-branch lesions. An online dynamic random method will be used to design and allocate hiding. Subjects will be randomly divided into the QSYQ group and the placebo group at a 1 : 1 ratio. We plan to use the clinical trial data management platform commissioned by the China-Japan Friendship Hospital to generate the random number and group number with the “YinRuiDa Randomization and Drug Management System.” Following provision of informed consent, the patient's name and ID number will be input into the “YinRuiDa Randomization and Drug Management System,” and a random number and group number will be generated. The patient number for each subject will remain the same throughout the study.

Drug random grouping: each patient will receive the drug or placebo 3 times, every 28 days (*n* = 99 copies per group; 1 copy = 1 dose for 28 days per person). SAS version 9.4 statistical software will be used to generate numbers 1–198, with each dose for 28 days per person being allocated one number.

#### 2.5.2. Blinding

The clinical trial data management platform of the China-Japan Friendship Hospital will be entrusted as the third party, and the “YinRuiDa Randomization and Drug Management System” will be used for grouping. Neither the subjects nor researchers will know the specific grouping results. To keep the study double-blind, QSYQ and placebo will be packaged in the same way, and the pills will be identical in shape, color, odor, and weight. Drug coding blindness takes one dose for 28 days per person as the unit, and each patient will be given drugs three times. Each drug will have a serial number, and the drug information hidden in each serial number will be entrusted to the third party for online storage.

#### 2.5.3. Unblinding

The first blinding will be performed after treatment; that is, all of the patients will be divided into groups A and B. After the end of the experiment, the blinding will be broken for the second time; that is, the blinding will be broken for group A or group B (QSYQ group or placebo control group). If serious adverse events (AES) occur, the results will be unblinded twice in two steps.

#### 2.5.4. Sample Size Calculation

After interventional treatment for patients with coronary heart disease (CHD), according to the reference literature, the difference in the amplitude of the increase in peak oxygen consumption was 3.33 mL/(min(/), the standard deviation was 3.65 mL/(min(/), the error of class I was 0.05, and the test efficiency was 90%. The following formula was used to calculate the sample size of each group:

(1) $$\begin{array}{c} n=\frac{2{\left(\mu_{\alpha }+\mu_{\beta }\right)}^{2}\sigma^{2}}{\delta^{2}}. \end{array}$$

The sample size of each group was 26; considering a 20% rate of shedding, 33 samples were calculated. According to the order of entering the group, 33 cases will be randomly divided into the QSYQ group and the blank placebo control group.

### 2.6. Interventions

#### 2.6.1. Drug Elution

The trial will involve drug elution for 1 week in the screening period before enrollment, that is, stopping other TCM used for CHD, including decoctions, Chinese patent medicine, and substitute tea.

#### 2.6.2. QSYQ Group

Standardized drugs combined with QSYQ will be administered for 12 weeks. The standardized drugs will include dual-antiplatelet, lipid-lowering, antihypertensive, hypoglycemic, and other symptomatic treatments; QSYQ is administered 1 bag at a time (0.5 g/bag), 3 times a day, taken 30 min after a meal.

#### 2.6.3. Blank Placebo Control Group

The patients will be treated with standardized Western medicine combined with placebo for 12 weeks. The standardized drugs will include dual antiplatelet, lipid-lowering, antihypertensive, hypoglycemic, and other symptomatic treatments; placebo is administered 1 bag at a time (0.5 g/bag), 3 times a day, taken 30 min after a meal.

### 2.7. Follow-Up

Following enrollment, all subjects will be followed up every 2 weeks, and the researchers set the content of each follow-up. After 8 weeks, all subjects will be followed up every 4 weeks until the end of the 12-week study. Follow-up will involve recording changes in TCM symptoms, Seattle scale score, and quality of life scale score.

## 3. Outcome

The primary outcome is the rate of change of VO_2_ peak. The secondary outcomes are as follows: (1) assessment of target organ damage, including changes in systolic and diastolic function indices (left ventricular ejection fraction, stroke output, left ventricular end-diastolic volume, *E*/*E*′, left atrial volume, etc.) in echocardiography observed before and after treatment; (2) symptom improvement evaluation using the TCM syndrome observation table (Table 2); Seattle scale and quality of life scale will be used to evaluate angina pectoris symptoms and quality of life; and (3) routine laboratory examination indicators, including routine bloods, liver and kidney function, and blood lipids.

### 3.1. Safety Assessment and Adverse Events Report

To evaluate the safety of Qishen Yiqi dripping pills and placebo, we will investigate abnormal changes in laboratory examination results before and after treatment and other adverse events. Once adverse events (including major adverse events) occur during the treatment, the occurrence time, clinical manifestations, treatment process and duration of adverse events, outcome, and the relationship with drugs will be recorded in detail on the case report form. Patients with laboratory abnormalities will be followed until the test results return to normal, to the preadministration level, or until it is determined that the adverse event is unrelated to the test drug. If a serious adverse event occurs, the Serious Adverse Event Form will be completed and reported to the Ethics Committee within 24 h.

### 3.2. Data Management

In this study, case report form (CRF) tables will be used to collect data. The subject information will not be directly recorded in the CRF but will be recorded on the original medical record of each subject, which will be retained as the original data. Medical and other records of subjects will be maintained by the investigator. These records should include the following: original data, copies of laboratory data, and results of other medical tests (e.g., electrocardiogram, etc.). The data in the CRF table will be taken from the original medical records and filled in by the researcher or the person designated by the researcher. The completeness and accuracy of the information will be ensured. The name of the person who made the change and the date of the change will be immediately recorded for any changes made to the CRF table. Upon completion of the CRF, it shall be submitted to the Data Statistics Center of the China-Japan Friendship Hospital in time for confirmation following raw data verification (SDV), data manager (DM) review, questioning, and other processing. Before data locking, the researcher will confirm the completeness and accuracy of data by signature. This trial has been registered in the “China Clinical Trial Registry” (http://www.chictr.org.cn/index.aspx) before the formal start of the trial. To facilitate follow-up tracing and verify the authenticity of the data, the study data related to the trial will be uploaded to the above website and made public at the end of the trial. The registration number is ChiCTR2000040838 (registration date is December 11, 2020).

### 3.3. Data Analyses

The international SAS version 9.4 statistical analysis software will be used for statistical analysis. The measurement data of each visit for different treatment groups will be statistically described by means of mean *u* standard deviation or median (minimum and maximum). Paired *t*-test will be used to compare the differences between the two groups. Changes before and after treatment in the two groups will be compared by analysis of variance or nonparametric test. The frequency (constituent ratio) will be used to describe the count data of each visit in different treatment groups. The changes before and after treatment in the two groups will be analyzed using the *χ*2 test, exact probability method, or nonparametric test. Except for special instructions, the bilateral test will be used to examine statistical significance, and *P* ≤ 0.05 will be used as the criterion for judging the significance of the difference.

### 3.4. Quality Control

Before the start of the clinical trial, all investigators in the clinical study will participate in Good Clinical Practice (GCP) training, including the GCP regulations and standard operating procedures. All investigators are trained in research protocols and procedures, will read and understand the content of the clinical study protocol, master the principles of GCP, unify the recording methods and judgment standards, and strictly follow the protocol. All participants in the test will have clear responsibilities, act according to the rules, and assume their own responsibilities.

After completing the CRF, it will be submitted to the data statistics center in time for data verification and data locking. Data verification will include manual verification and data verification meetings. The researcher or the personnel designated by the researcher shall correct any inconsistent data found in the verification. Following confirmation that the CRF data are correct at the verification meeting, the main investigator, sponsor, and statistical analyst will sign the relevant documents to lock the test data. The locked data are not allowed to be changed in principle. Data problems discovered after a data lock will be confirmed for revision and documented in accordance with the provisions of the data management plan.

### 3.5. Trial Status

This is an ongoing experiment. By December 2020, the trial had passed the ethical review and clinical study registration, and the project kickoff meeting was successfully held. Subjects are currently being recruited for this study.

## 4. Discussion

PCI can rapidly open severe stenosis and occlusion vessels, and is currently one of the main methods of revascularization [22]. However, according to the data from the British ORBITA study [23], PCI has no significant impact on angina attack and exercise endurance in patients with stable coronary heart disease. Additionally, PCI may lead to myocardial reperfusion injury, local vascular endothelial injury, and in-stent restenosis, all of which may induce the recurrence of cardiovascular events in serious cases [24–26]. Therefore, CR treatment is an urgent need. At present, exercise rehabilitation is widely used in patients with coronary heart disease and other cardiovascular diseases, and has become an important component of the research on CR after PCI [27]. However, patient participation is low, and it is difficult to adhere to it for a long time, which greatly reduces the effectiveness of CR.

The advantages of TCM in CR after PCI have gradually emerged in recent years, and the integration of traditional Chinese and Western medicine has achieved remarkable curative effects [28, 29]. Additionally, for patients who are unsuitable for exercise rehabilitation after PCI, the treatment of integrated Chinese and Western medicine shows great value in improving their prognosis. At present, QSYQ have been incorporated into PCI in TCM diagnosis and treatment of postoperative chest pain expert consensus (2014) and into the expert consensus on Phase 1 CR of integrated traditional Chinese and Western Medicine (2016). In this study, patients with stable angina pectoris caused by Qi deficiency and blood stasis after interventional coronary heart disease (CHD) were selected as the research objects. Using a randomized, double-blind, placebo-controlled method, the peak oxygen consumption was taken as the main efficacy evaluation index to study the effect of Qishen Yiqi dripping pills on exercise endurance and quality of life of patients after PCI.

The main hypotheses of this study are as follows: (1) most patients with CHD after interventional surgery suffer from Qi deficiency and blood stasis, and the long-term application of compound TCM to replenish Qi and activate blood circulation improves exercise endurance, reduces the risk of angina pectoris, reduces the rate of readmission, and improves quality of life; and (2) the effect of integrated traditional Chinese and Western medicine on CR is better than that of traditional exercise rehabilitation. The innovation points are mainly reflected in the following three aspects:

- A standardized measurement is needed to effectively assess changes in exercise endurance before and after treatment. We chose the rate of change of peak oxygen consumption (PeakVO_2_), the gold standard of exercise endurance, as the main result. Considering the indications and contraindications of cardiopulmonary exercise, patients with stable angina pectoris after coronary heart disease intervention were selected as the research object to avoid the occurrence of cardiovascular events, such as acute myocardial infarction, unstable angina pectoris, and malignant arrhythmias, caused by exercise.
- This program can give full play to the advantages of syndrome differentiation and treatment, early intervention, and multitarget of TCM and supplement the deficiency of simple rehabilitation methods of Western medicine. After syndrome differentiation, we will include patients with stable angina pectoris of Qi deficiency and blood stasis type. On the basis of conventional Western medicine treatment, QSYQ and placebo will be given orally.
- The study protocol was designed according to international clinical trial principles. A blank placebo randomized double-blind control was designed to ensure the authenticity and reliability of the research data. To reduce the selection bias caused by the number of enrolled cases, we used stratified randomization. Two stratification factors, “sex” and “number of disaffected vessels,” were set for randomization, with the purpose of reducing type I errors, improving the assurance of small sample trials and ensuring the balance of sample distribution between groups.

## Figures and Tables

**Table 1:** Diagnostic criteria of Qi deficiency and blood stasis syndrome.

	Signs and symptoms
Symptoms	A: fatigue, shortness of breath, and physical weakness, with aggravation after activity; B: purple and dark lip color
Tongue presentation	The tongue is dark or has ecchymosis and ecchymosis; the sublingual vein is tortuous, dilated, and dark purple
Pulse condition	Weak pulse, astringent pulse
Diagnosis	One of A and one of B; or one of A and one abnormal tongue presentation pulse image for clinical diagnosis reference

**Table 2:** TCM syndromes observation table.

Definition	Meaning
Clear effect	Clinical symptoms and signs were significantly improved, and the syndrome score was reduced by 70%
Valid	Clinical symptoms and signs were improved, and the syndrome score decreased by 30%
Invalid	Clinical symptoms and signs were not significantly improved, or even aggravated, and the syndrome score was reduced by <30%
Aggravation of symptoms	Clinical symptoms and signs were aggravated, and the syndrome score decreased by < 0

## Data Availability

In order to facilitate follow-up tracing and verify the results of an article, the study data related to the trial will be uploaded and made public at the end of the trial. The data that support the findings of this study are openly available in the “China Clinical Trial Registry” (http://www.chictr.org.cn). The registration number is ChiCTR2000040838 (registration date is December 11, 2020).
